# The design and statistical aspects of VIETNARMS: a strategic post-licensing trial of multiple oral direct-acting antiviral hepatitis C treatment strategies in Vietnam

**DOI:** 10.1186/s13063-020-04350-x

**Published:** 2020-05-18

**Authors:** Leanne McCabe, Ian R. White, Nguyen Van Vinh Chau, Eleanor Barnes, Sarah L. Pett, Graham S. Cooke, A. Sarah Walker, on behalf of SEARCH investigators, Eleanor Barnes, Graham S. Cooke, Jeremy N. Day, Nguyen Thanh Dung, Barnaby Flower, Tim Hallett, Le Manh Hung, Evelyne Kestelyn, Dao Bach Khoa, Leanne McCabe, Sarah L. Pett, Le Thanh Phuong, Motiur Rahman, Joel Tarning, Hugo C. Turner, Guy E. Thwaites, Nguyen Van Vinh Chau, A. Sarah Walker, Nicholas J. White

**Affiliations:** 1 Medical Research Council Clinical Trials Unit at University College London, 90 High Holborn, WC1V 6LJ London, UK; 2 Hospital for Tropical Diseases, 764 Vo Van Kiet, Ho Chi Minh City, Vietnam; 3 Oxford University, South Parks Road, OX1 3SY Oxford, UK; 4 Imperial College London, SW7 2AZ London, Kensington UK

**Keywords:** Adaptive design, Bayesian methods, Clinical trial, Hepatitis C, Interim analyses, Multi-arm, Trial design

## Abstract

**Background:**

Eliminating hepatitis C is hampered by the costs of direct-acting antiviral treatment and the need to treat hard-to-reach populations. Access could be widened by shortening or simplifying treatment, but limited research means it is unclear which approaches could achieve sufficiently high cure rates to be acceptable. We present the statistical aspects of a multi-arm trial designed to test multiple strategies simultaneously and a monitoring mechanism to detect and stop individual randomly assigned groups with unacceptably low cure rates quickly.

**Methods:**

The VIETNARMS trial will factorially randomly assign patients to two drug regimens, three treatment-shortening strategies or control, and adjunctive ribavirin or no adjunctive ribavirin with shortening strategies (14 randomly assigned groups). We will use Bayesian monitoring at interim analyses to detect and stop recruitment into unsuccessful strategies, defined by more than 0.95 posterior probability that the true cure rate is less than 90% for the individual randomly assigned group (non-comparative). Final comparisons will be non-inferiority for regimens (margin 5%) and strategies (margin 10%) and superiority for adjunctive ribavirin. Here, we tested the operating characteristics of the stopping guideline for individual randomly assigned groups, planned interim analysis timings and explored power at the final analysis.

**Results:**

A beta (4.5, 0.5) prior for the true cure rate produces less than 0.05 probability of incorrectly stopping an individual randomly assigned group with a true cure rate of more than 90%. Groups with very low cure rates (<60%) are very likely (>0.9 probability) to stop after about 25% of patients are recruited. Groups with moderately low cure rates (80%) are likely to stop (0.7 probability) before overall recruitment finishes. Interim analyses 7, 10, 13 and 18 months after recruitment commences provide good probabilities of stopping inferior individual randomly assigned groups. For an overall true cure rate of 95%, power is more than 90% to confirm non-inferiority in the regimen and strategy comparisons, regardless of the control cure rate, and to detect a 5% absolute difference in the ribavirin comparison.

**Conclusions:**

The operating characteristics of the stopping guideline are appropriate, and interim analyses can be timed to detect individual randomly assigned groups that are highly likely to have suboptimal performance at various stages. Therefore, our design is suitable for evaluating treatment-shortening or -simplifying strategies.

**Trial registration:**

ISRCTN registry: ISRCTN61522291. Registered on 4 October 2019.

## Background

Oral direct-acting antivirals (DAAs) have transformed the treatment of hepatitis C virus (HCV). Compared with historical injectable interferon-based treatment of 6–12 months, they are more effective and better tolerated and offer shorter durations of therapy (8–12 weeks) [1]. However, access to treatment is still limited by costs, particularly in low-income countries where the patient pays at least in part [2]. Furthermore, achieving the World Health Organization (WHO) target for elimination of viral hepatitis as a public health threat by 2030 [3] will require curing hard-to-reach populations, including the homeless, drug users and prisoners who still find adherence challenging. Strategies designed to reduce drug exposure while still achieving HCV cure could further widen treatment access, but to date there has been limited research into shortening and simplifying HCV treatment with DAAs.

Previous very small studies assessing shortened DAA treatment have found higher cure rates when treatment length is guided by early response to treatment [4–6] or when adding adjunctive therapies, such as pegylated interferon (PEG-IFN) [7], to DAAs. Outside of HCV treatment, drug-sparing strategies that allow intermittent dosing are widely used in the treatment of tuberculosis [8], allowing patients who may not comply with daily treatment to access supervised treatment. Although these approaches may also be successful in HCV, no studies are currently assessing such a strategy with DAAs. There has also been little research into the use of DAAs in genotype 6, a strain that is most prevalent in Vietnam and surrounding countries (~50%) but uncommon elsewhere (<5%) [9]. Several small studies have shown very high cure rates with DAAs in this genotype [10–13], as in other genotypes, but these have been mostly limited in regimen and to standard-length courses. Finally, three pan-genotypic regimens (sofosbuvir/velpatasvir, sofosbuvir/daclatasvir and pibrentasvir/glecaprevir) are recommended by the most recent WHO guidelines [14], but to date there has been no direct randomised comparison of these regimens in any genotype.

As there are very few data to inform optimal ways to shorten HCV treatment, especially in genotype 6, it is possible that any proposed shortening strategy will fail. Therefore, trial designs that include many different options whilst allowing for the early stopping of unsuccessful treatments in order to focus on more successful treatments are essential, both for trial efficiency and to protect patients. Two trial designs that incorporate both of these aspects are factorial trials and multi-arm multi-stage (MAMS) trials. Both designs allow for greater efficiency in trials by reducing the number of patients required and shortening the time needed to test multiple interventions compared with sequential trials of individual interventions [15, 16]. Where an interaction between interventions being tested is anticipated, MAMS trials are preferred as this increases power to detect them [17]. However, the timing and maximum number of interim analyses within MAMS trials generally have to be pre-specified [18] or otherwise controlled with an alpha-spending function, which can be computationally difficult, particularly as the complexity of the design increases [19]. Therefore, MAMS designs may be less suitable for interventions where the effect on outcomes is unknown and the interim analysis schedule may have to be altered, at least where *a priori* interactions are not expected.

Data monitoring and stopping guidelines are most commonly framed within a frequentist framework with guidelines based on *P* values or conditional power. In the planning of interim analyses, it is generally accepted that care must be taken to control type I error, which can limit the ability to change the monitoring schedule to adapt to accumulating data, which may lead to delays in stopping unsuccessful treatments (e.g., if strict guidelines such as Haybittle–Peto (*P* <0.001) are used). Although strict error control may not always be necessary in a trial using frequentist methods [20], a more flexible approach to monitoring can be easier to implement and justify by using a Bayesian approach, which allows for stopping guidelines that are based on directly interpretable probabilities, particularly in complex multi-arm trials [21–24]. Incorporating Bayesian monitoring within a multi-arm factorial trial can allow for a flexible monitoring schedule to test multiple strategies and detect inferior ones quickly. Previous designs have looked at multi-arm trials [25, 26] but have not examined the impact of factorial randomisation.

Here, we present the statistical aspects of the design of a multi-arm factorial trial (to be conducted in Vietnam) that aims to find efficacious drug-sparing treatment strategies that will allow access to HCV treatment to be widened, and our particular focus is on increasing evidence on treatment of genotype 6.

## Methods

### Trial design

**Fig. 1:**
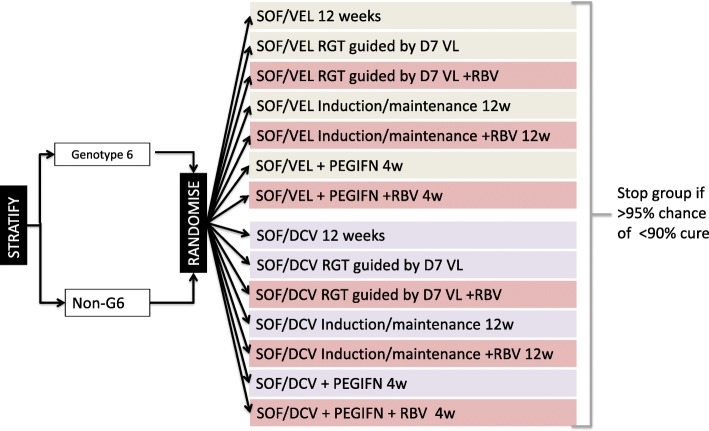
Trial schema. *Abbreviations*: *PEG-IFN* pegylated interferon, *RBV* ribavirin, *RGT* response-guided therapy, *SOF/DCV* sofosbuvir/daclatasvir, *SOF/VEL* sofosbuvir/velpatasvir, *VL* viral load.

VIETNARMS is a parallel-group open-label factorial trial (ISRCTN61522291); 1092 patients will be factorially randomly assigned 1:1 to two different WHO-recommended dual DAA regimens (sofosbuvir/velpatasvir versus sofosbuvir/daclatasvir), 1:2:2:2 to the standard licensed 12-week treatment versus 4-week treatment with PEG-IFN + DAA versus 4- to 12-week response-guided therapy (RGT) versus 12-week treatment using an induction/maintenance approach, and (if not randomly assigned to standard 12-week treatment) 1:1 to adjunctive ribavirin versus no ribavirin for the duration of their DAA treatment (Fig. 1). Randomisation will be stratified by genotype 6 versus all other genotypes. Patients randomly assigned to the PEG-IFN strategy will receive DAAs for 4 weeks with weekly PEG-IFN for 4 weeks starting at day 7. Treatment length for those randomly assigned to RGT will be determined by HCV viral load (VL) at day 7 and based on predicted viral kinetics [27]: those with VL of less than the lower limit of quantification (LLOQ) will receive 4 weeks of treatment, those with VL LLOQ-250 IU/mL will receive 8 weeks and all others will receive 12 weeks. Patients randomly assigned to induction/maintenance will receive 12 weeks of treatment: 2 weeks of daily treatment (induction phase) followed by 10 weeks of 5-day treatment per week, taking weekends off from the first weekend following their full 2-week treatment. Each strategy reduces DAA exposure and has other benefits, such as compatibility with directly observed therapy programmes (as used for tuberculosis) but with other additional costs (Table 1). Within the trial, any patient not achieving cure with their first-line treatment will receive 12 weeks of retreatment with the alternate drug regimen to the one they were originally randomly assigned plus ribavirin.

**Table 1:** Components of each shortening strategy and cost differences compared with standard 12-week treatment

Shortening strategy	Resource use of providing treatment				Difference in cost compared with standard 12-week treatment
	Mean weeks of DAA	Number of PEG-IFN injections	Number of visits required	Number of VL tests	
Standard 12-week treatment	12	0	3^a^	1^a^	–
PEG-IFN + DAA (4 weeks’ treatment, weekly PEG-IFN)	4	4	6	1	-8x(weekly drug cost) + 4x(interferon cost) + 3x(visit cost)
Response-guided therapy (4, 8, or 12 weeks’ treatment)	9.6^b^	0	4	2	−3.4x(weekly drug cost) + 1x(visit cost) +1x(VL cost)
Induction/maintenance (7 days/week for 2 weeks, then 5 days/week)	9.14	0	4^c^	1	−3.86x(weekly drug cost) + 1x(visit cost)

*Abbreviations*: *DAA* direct-acting antiviral, *PEG-IFN* pegylated-interferon, *VL* viral load.

^a^ Assuming minimum visits at treatment initiation, end of treatment, end of treatment plus 12 weeks (where VL is done once to assess cure)

^b^ Assuming 1:3:1 receiving 4:8:12 weeks of treatment. One extra visit and VL at day 7 to assess initial VL response

^c^ Assuming one extra visit at week 2 when move from induction to maintenance phase

### Primary endpoint

The primary endpoint is sustained virologic response (SVR12), namely virological cure on first-line therapy defined as plasma HCV VL of less than LLOQ 12 weeks after the end of first-line treatment (EOT + 12) without prior failure. Failure is defined as either two consecutive HCV VLs of more than LLOQ after two consecutive HCV VLs of less than LLOQ with the latter confirmatory VL of more than 2000 IU/mL or two consecutive HCV VLs of more than 1 log_10_ increase above HCV nadir on treatment and of more than 2000 IU/mL (either definition being met whilst on treatment or after finishing treatment during follow-up). The primary outcome is binary, and all observed endpoints are either SVR12 or treatment failure.

These failure criteria identify patients who, were they not to receive retreatment before EOT + 12 weeks, would definitively have HCV VL greater than LLOQ at 12 weeks post-EOT and hence would be considered failures in the primary endpoint. However, for ethical reasons, within the trial such patients will be offered retreatment as soon as they are definitively identified as having failed first-line treatment. Failure is defined using a higher threshold than the LLOQ because patients have been observed to achieve cure despite having low-level viraemia at EOT or shortly after and so they do not need retreatment to achieve cure on first-line treatment. In practice, any participant with low-level viraemia of less than 2000 IU/mL either cures or VL rises above this level. This will be carefully reviewed by the independent data monitoring committee (DMC). This is the same definition as used in the UK STOP-HCV-1 trial (ISRCTN37915093) and SEARCH-1 trial in Vietnam (ISRCTN17100273), where the DMC has similarly reviewed individual patient VL trajectories.

### Monitoring

For the strategies to be viable outside the trial, first-line cure rates need to be high (>90%). The design of the trial therefore allows for failing groups to be stopped early at any time and subsequent patients to be randomly assigned to more successful groups. Individual performance of groups receiving shortening strategies will be monitored during recruitment by an independent DMC that will make decisions on whether a group should be stopped. Groups receiving standard 12-week treatment will not be monitored as this is the licensed duration with cure rates of more than 90% [28, 29]. Interim analyses will not be comparative as the aim of monitoring is not to find the best strategy but to find any strategy that meets a minimum acceptable cure rate that may also be non-inferior to standard treatment as different strategies may benefit different patient populations.

Analyses of cure rates will follow the Bayesian paradigm to allow the probability of the true cure rate being below different thresholds to be calculated: recruitment into a group will stop if there is a greater than 0.95 posterior probability of the true cure rate being less than 90% (Pr(true cure rate <0.9|x) >0.95, where x is the data currently observed). The primary monitoring is combined across genotypes; if the combined group reaches the stopping guideline, each genotype stratum will be tested separately and the DMC will have the discretion to stop only those strata reaching the stopping criteria. Differences in stopping groups across strata are likely to occur only when there are extreme differences in the cure rates between the strata, which is not expected, and so the operating characteristics of the trial are based on stopping combined strata only. If neither stratum reaches the stopping criteria despite the combined strata doing so, it will be at the discretion of the DMC whether to stop recruitment into the stratum or group.

At interim analyses, there is greater uncertainty about the performance of the shortening strategies. Therefore, when the prior was determined, it was assumed that one strategy would fail completely such that all four groups receiving that strategy, of a total of 12 tested, would meet the stopping guideline. As each individual outcome is assumed to be Bernoulli-distributed and therefore distribution of all outcomes is binomial, a beta prior was chosen as this is the conjugate prior for the binomial distribution. The mean of the prior was fixed at 0.9, and the effective sample size of the prior was varied until a distribution was found such that there was a roughly 0.33 probability of a cure rate of less than 90%; roughly 0.33 probability was chosen as 4 out of 12 randomly assigned groups are expected to fail and therefore have a true cure rate of less than 90%. The prior chosen was beta (4.5, 0.5) with mean 0.9, variance 0.015 and a 0.34 probability of a cure rate of less than 90%. The relatively low precision of the prior will allow greater influence of the data in the posterior distribution. If the stopping guideline is met, sensitivity analyses using priors informed by observed cure rates in other randomly assigned groups or strata will be performed and will be provided to the DMC to help inform their decision to stop a group.

### Sample size

The sample size of the trial was derived on the basis of the null hypothesis that the true cure rate was 90% in each group compared with an alternative, unacceptably low, cure rate of 70%; assessment of power at the final analysis given this sample size is discussed later. For a single stratum within a single randomly assigned group, given 90% power and one-sided alpha of 0.05, and 5% loss to follow-up by EOT + 12, 39 patients would be required per group to exclude the cure rate being lower than 70% based on a single-group test. There are 14 groups and two genotype strata, giving a total sample size of 1092 patients (39*14*2). For final comparisons at trial closure, the null hypothesis is that all groups will achieve the more than 90% cure target and be included in the final analysis. There will then be 546 per group for the regimen comparison (non-inferiority; comparing the two WHO-recommended drug regimens against each other), 156 in the control group and 312 per intervention group for the strategy comparison (non-inferiority; comparing each of the three treatment-shortening strategies versus the licensed 12-week control duration), and 468 per group for the ribavirin comparison (superiority; comparing each treatment-shortening strategy with and without ribavirin). The total sample size is fixed at 1092 patients; if individual groups are stopped early, any subsequent patients will be randomly assigned to open groups where possible (depending on the delay between randomisation, identification of primary endpoints and interim analyses), so numbers in each fully recruited group may be higher. This is appropriate in a pragmatic trial, where the goal is to maximise information gained about many different strategic approaches to treatment rather than to minimise sample size per se.

The choice of the non-inferiority margin was based on clinical judgement and the size of margins used in other trials of anti-infectives with relatively low failure rates such as community-acquired pneumonia [30]. In particular, a smaller 5% margin is chosen for the drug comparisons because in practice they are likely to have similar advantages and disadvantages. In contrast, the different drug-sparing strategies have a variety of different advantages and disadvantages (in terms of additional visits vs. less drug vs. different drugs vs. weekends off; Table 1) which could be differentially balanced against overall cure rates, particularly considering impact on health-care provision (e.g., through directly supervised therapy). Thus, a greater non-inferiority margin (here 10%) is relevant to drug-shortening because the potential benefit in terms of numbers treated for the same fixed budget is much greater. Regulatory guidance recommends that non-inferiority margins be chosen to ensure that the difference between an intervention and the active control (here 12 weeks’ duration) would not exceed that between the active control group and a hypothetical placebo (or other standard control group, here the previous standard of care of 12–48 weeks of PEG-IFN) [30]. As cure rates with 12–48 weeks of PEG-IFN were about 70% in genotype 6 [31], with similar or lower cure rates in other genotypes, and as we expect more than 90% SVR12 in all groups, our non-inferiority margins of both 5% for the regimen comparison and 10% for the strategy comparison would ensure this.

From initial power calculations based on all 14 randomly assigned groups, assuming that the overall cure rate is 95%, the fixed total sample size of 1092 patients above provides 97% power to demonstrate non-inferiority between drug regimens based on a 5% margin and 96% power to demonstrate non-inferiority between shortening strategies based on a 10% margin, both with one-sided alpha of 0.05. For superiority comparisons (conducted for ribavirin and any comparison that meets non-inferiority above) and two-sided alpha of 0.05, these numbers provide more than 90% power to detect absolute differences in SVR12 of 5% for regimen or ribavirin comparisons and more than 80% power to detect absolute differences in SVR12 of 7% or more for the strategy comparisons.

### Statistical analysis

The final analysis will estimate risk differences between groups using marginal effects after logistic regression. The model will include all main randomised effects and strata and will test interactions between all randomisations (Supplementary Methods, Additional file 1). Interactions will be included in the final model only if the 95% credible interval for the interaction term excludes no effect (*P* <0.05 for frequentist analyses). The interaction between regimen and strategy will include all levels of strategy. Owing to the partial factorial randomisation, the interaction between ribavirin and strategy will not include the standard treatment length strategy. Comparisons of regimens and of strategies will be non-inferiority analyses, and the ribavirin comparison will be a superiority analysis. Primary analysis will be intention-to-treat using Bayesian methods and 90% credible intervals. Secondary analyses will consider per-protocol populations, frequentist methods, and 95% credible and confidence intervals.

Analysis priors for the final analysis are listed in Table 2; these differ from the monitoring priors as the aim of monitoring is only to identify poorly performing groups and not to compare the randomly assigned groups. The control cure rate analysis prior is beta (4.75, 0.25), which has a mean of 0.95 and a variance derived as for the monitoring priors. This mean was derived from previous research into the trial drug regimens [28, 29]. Sensitivity analyses will use a range of informative priors reflecting plausible belief in the clinical community. Sceptical analysis prior distributions were chosen with means corresponding to the null hypothesis for each randomisation and enthusiastic analysis priors with means y greater than this, where y is the non-inferiority margin or absolute difference specified in the power calculations. The variances were arbitrarily set such that 90% of the prior distribution is within ± y around the mean to reflect the strength of the belief in the mean effect. Thus, for example, the risk difference for the drug regimen comparison has the sceptical analysis prior centred on −5% (the null hypothesis for the non-inferiority comparison) with 90% limits ± 5%, giving a 0.05 probability that the cure rate will be 10% worse and a 0.05 probability the cure rate will increase (i.e., be more than 0%) (sceptical priors in each direction will be used for the regimen comparisons). The enthusiastic analysis prior is centred on 0% with 90% limits ± 5%, giving a 0.05 probability that the cure rate will be 5% worse and a 0.05 probability that it will be 5% better with one regimen than the other.

**Table 2:** Priors to be used in final analysis

	Primary analysis: uninformative	Sensitivity analyses	
		Sceptical	Enthusiastic
Control cure rate	Beta(4.75, 0.25)	Beta(4.75, 0.25)	Beta(4.75, 0.25)
Regimen comparison	N(0, 10,000)	N(−0.05, 0.009)	N(0, 0.009)
Strategy comparison	N(0, 10,000)	N(−0.1, 0.0036)	N(0, 0.0036)
Ribavirin comparison	N(0, 10,000)	N(0, 0.009)	N(0.05, 0.009)

The beta prior for the control cure rate has mean 0.95 and variance 0.008

The model will also be adjusted for genotype, which will have the prior N (0, 10,000) in all analyses

N(m,v) is the normal prior with mean m and variance v

To define the performance characteristics of the proposed stopping guideline, posterior probabilities of cure rates and the probability of stopping groups at each number of outcomes were calculated analytically using beta and binomial distributions respectively. Timings of interim analyses were determined by applying the probabilities of stopping groups to a projected recruitment schedule. The average probability of stopping a genuinely inferior group was estimated by integrating the probability of stopping a group with respect to the monitoring prior beta (4.5, 0.5) over cure rates between 60% and 90%. The lower bound was determined from previous studies testing strategies most similar to those in VIETNARMS which have reported cure rates of more than 90% with lower confidence interval bounds of more than 60% [6, 7]. In studies with cure rates of less than 60%, all patients received shortened therapies, regardless of their HCV VL, and did not receive adjunctive drugs [32, 33]; therefore, they were not considered relevant to this analysis, although power would be even greater to stop such a group. Cure rates above 90% were not considered since groups with these cure rates should not be stopped and so do not affect timing of the analyses. Simulations of 5000 datasets with outcomes taken from binomial distributions were used to determine the overall probability of stopping a group, the cumulative probability of stopping groups at specified analysis time points, and to estimate power after being analysed using marginal effects after logistic regressions with a model containing all randomised comparisons, as described above. Predictive probabilities (the probability of achieving a success at the end of the trial) were calculated analytically using the beta-binomial distribution in R 3.5.1. All other analyses were performed by using Stata version 15.1.

## Results and Discussion

### Characteristics of Bayesian stopping guideline for individual groups

The minimum number of failures required to satisfy the stopping criteria for the main monitoring beta (4.5, 0.5) prior and for each number of analysed patients is listed in Table 3. The probability of stopping a group is then the probability of observing the required number of failures in the group. When the true cure rate is 90%, the probability of incorrectly stopping a group is always less than 0.05 and this decreases as the true cure rate increases. It is expected from the specification of the stopping guideline that when the true cure rate is equal to the mean of the prior, the probability of stopping a group is 0.05, so the probability of incorrectly stopping a group is always maintained below the correct level. The calculated probability is not exactly 0.05 and differs depending on the number of patients analysed due to the discrete nature of the outcome.

**Table 3:** Number of failures needed to stop a drug strategy group for each number of analysed patients

Analysed patients	Minimum number of observed failures needed to stop group^a^	Maximum probability of stopping group if true cure rate equals:		Minimum probability of stopping group if true cure rate equals:			
		90%	95%	90%	80%	70%	60%
3–7	3	0.026	0.004	0.001	0.008	0.027	0.064
8–13	4	0.034	0.003	0.005	0.056	0.194	0.406
14–20	5	0.043	0.003	0.009	0.130	0.416	0.721
21–26	6	0.040	0.002	0.014	0.231	0.637	0.904
27–33	7	0.042	0.001	0.015	0.287	0.744	0.958
34–39^b^	8	0.037	0.001	0.017	0.367	0.844	0.986
40–41^b^	8	0.048	0.001	0.042	0.563	0.945	0.998
42–48	9	0.046	0.001	0.021	0.469	0.920	0.997
49–55	10	0.044	0.0004	0.022	0.528	0.952	0.999
56–63	11	0.047	0.0003	0.021	0.580	0.971	1.000
64–71	12	0.048	0.0002	0.023	0.648	0.985	1.000
72–78	13	0.045	0.0001	0.025	0.705	0.993	1.000

Groups will recruit a minimum of 78 patients with 39 patients in each stratum if recruitment is not stopped into that group. If recruitment is stopped into one group, the maximum number of patients in each other group and the other stratum for that group will be higher

Maximum and minimum probabilities are for the range of analysed patients

^a^ More than 0.95 posterior probability of the true cure rate being less than 90% (Pr(true cure rate < 0.9|x) > 0.95, where x is the data currently observed, with the beta (4.5, 0.5) prior which has mean = 0.9 and variance = 0.015

^b^ These rows not pooled despite the same number of minimum failures to provide information about the fully recruited strata (*n* = 39)

For small numbers of analysed patients, a larger proportion of failures are required to stop a group, increasing from a minimum of 17% to 100% of those analysed; therefore, the probability of stopping a group incorrectly early in recruitment is also smaller as there is a smaller chance of observing greater proportions of failures regardless of the true cure rate. This protects against groups being stopped erroneously because of a high concentration of failures amongst the initial patients reaching EOT + 12, and if the two strata within a group share the same true cure rate, then it is unlikely that only one will reach the stopping threshold.

Groups with the lowest cure rates considered plausible (60%) are highly likely to reach the stopping criteria quickly (>90% chance of stopping after analysing 21–26 patients). Groups with moderately low cure rates (<80%) are also likely to be stopped before recruitment ends. However, groups with true cure rates slightly under 90% are unlikely to be stopped (results not shown). A low chance of stopping a group just below the target cure rate might be considered unacceptable in futility stopping guidelines in other situations, but the target of 90% is largely arbitrary and there may be interest in strategies that have a slightly lower cure rate if they are able to expand treatment access to difficult-to-reach populations. Increasing the probability of stopping groups with cure rates just below 90% would lead to a greater chance of incorrectly stopping groups with cure rates of more than 90% and it is considered more important to retain these than to stop groups with slightly lower cure rates. Additionally, any other stopping guideline would similarly be unable to discriminate between these cure rates without a very large sample size. The probability of incorrectly not stopping a group rapidly decreases as the true cure rate decreases.

The overall probability of stopping a group, and therefore making a correct decision or incorrect decision to stop recruitment into a group (again analogous to the frequentist concepts of power and type I error), shows results similar to those given above (Supplementary Table 1, Additional file 1). For cure rates of more than 90%, the probability of incorrectly stopping a group is always maintained less than 0.05, and for cure rates of less than 90%, there is a high probability of correctly stopping a group, and almost all groups are stopped when the true cure rate is not more than 70%. The probability of incorrectly not stopping groups with cure rates of about 80% is 12%, and for cure rates of 90%, a group is far more likely not to be stopped than correctly stopped. However, as described above, for our design this is not as much of a concern as it may be for other trial designs because the aim of the monitoring is to stop clearly inferior regimens rather than those close to the arbitrary 90% threshold.

### Timing of interim analyses

**Fig. 2:**
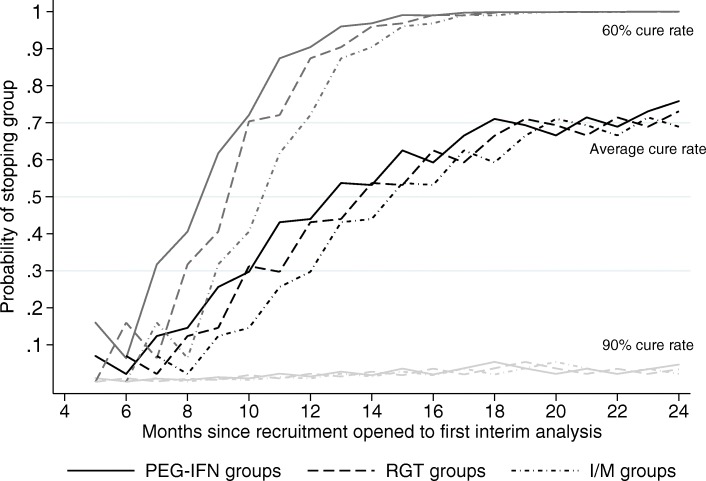
Initial probability of stopping groups over an estimated recruitment schedule for various true cure rates. The average true cure rate is uniformly distributed over 60–90%. Probabilities are calculated assuming no previous interim analysis. The total numbers of patients recruited at each month, of a total target of 1092, are 113 at 5 months, 153 at 6, 205 at 7, 257 at 8, 309 at 9, 361 at 10, 413 at 11, 465 at 12, 517 at 13, 569 at 14, 621 at 15, 673 at 16, 725 at 17, 777 at 18, 829 at 19, 881 at 20, 933 at 21, 985 at 22, 1037 at 23 and 1092 at 24. See Supplementary Figure 1, Additional file 1 for the cumulative probability of stopping a group.

Interim analyses need sufficient numbers of patients at EOT + 12 to give a reasonable probability of stopping a genuinely inferior group. Therefore, it was decided to perform analyses after the first month such that at least one inferior group has a 0.3, 0.5 or 0.7 average probability of being stopped were this to be the first interim analysis, assuming that cure rates are uniformly distributed on (0.6, 0.9) and given projected recruitment (Supplementary Table 2, Additional file 1). An average probability is used to reflect the uncertainty about the true cure rates; for low cure rates, the probability of stopping a group can be substantially higher (Fig. 2). An additional analysis before these thresholds will allow for any groups with a very low cure rate below the anticipated minimum of 60% to be detected early despite the very small probability of detecting a group with a cure rate between 60% and 90%.

Four analysis time points were chosen to provide multiple opportunities to detect failing groups while allowing adequate time between analyses for the accrual of patients and outcome data and preventing an unnecessary burden on time and resources needed for analyses and subsequent DMC meetings. The highest probability threshold (0.7) is determined by the maximum average cure rate of the genuinely inferior groups and by the recruitment schedule, as analyses need to be performed sufficiently early enough to gain the benefit of randomly assigning remaining patients to the other groups. The other thresholds (0.3, 0.5) were evenly spaced across the probabilities of stopping a group with an average cure rate, taking into consideration the first, early DMC meeting not based on these probabilities.

Therefore, considering the underlying projected recruitment, we expect the interim analyses to take place after 7, 10, 13 and 18 months. The number of patients in each group and the probability of stopping an inferior group of each strategy type at these analyses are listed in Table 4. By assessing the cumulative probability of stopping a group (Supplementary Fig. 1, Additional file 1), our schedule provides a balance between having more frequent meetings, which have a lower probability of stopping groups at any individual meeting but allow for earlier detection of poorly performing groups, and less frequent meetings, which have a higher probability of stopping a group but its low performance is detected later. The exception to this is having analyses every month but this schedule is impractical because of the resources required for an interim analysis.

**Table 4:** Timing of interim analyses

	First	Second	Third	Fourth
Months since recruitment started	7	10	13	18
Total recruited	205	361	517	777
Total at EOT + 12 weeks	44	144	286	533
At EOT + 12 weeks in each:				
PEG-IFN group	5	14	24	42
RGT group	3	11	21	39
Induction/maintenance group	2	8	17	35
Average probability genuinely inferior group will be stopped^a^				
PEG-IFN groups	0.124	0.297	0.537	0.710
RGT groups	0.021	0.313	0.440	0.665
Induction/maintenance groups	0.070	0.150	0.431	0.593

*Abbreviations*: *EOT + 12* 12 weeks after the end of treatment, *PEG-IFN* pegylated-interferon, *RGT* response-guided therapy

^a^ Assuming true cure rate uniformly distributed over 60–90%

As this is only projected recruitment, sensitivity analyses were performed to examine the effect of faster or slower recruitment (Supplementary Table 3, Additional file 1). These indicate that changes to the recruitment schedule alter only the timing of the analyses; the number of patients in each analysis differs by less than the estimated number recruited in 1 month. Therefore, if there are significant delays in recruitment, interim analyses will be timed such that they include a similar number of patients at EOT + 12 to that in the expected schedule. Sensitivity analyses also explored changing the lower bound of the distribution over which cure rates of genuinely inferior groups are assumed to be distributed to below 60% (Supplementary Table 4, Additional file 1) but this had minimal effect on the timing of initial interim analyses. There were greater differences in the timings of the last analyses, but the timing of this analysis is the most flexible and can be determined on the basis of observed rather than assumed true cure rates.

### Power for final analysis

Given the lack of knowledge of the standard 12-week cure rates for this population (anticipated 50% of patients with genotype 6) and how cure rates in the shortened treatment with ribavirin groups will compare to these and to shortened treatment without ribavirin, there is uncertainty regarding the overall power for the final analysis even if the overall cure rate is 95% (Table 5). If we assume equality between regimens and a 5% absolute difference for ribavirin, then these constraints mean that the cure rates in each group are completely determined by the difference between the shortening strategies. Non-inferiority can exist between the standard duration group and the pooled shortening strategy groups, the shortening strategy without ribavirin groups, or the shortening strategy with ribavirin groups, meaning that the shortening strategy with ribavirin groups can have cure rates 2.5% higher than, 5% higher than or equal to the standard duration groups respectively. These alternatives are shown in different columns of Table 5.

**Table 5:** Group-specific cure rates and power for 1092 patients with an overall 95% cure rate

	Ribavirin group cure rate compared with 12 week standard treatment cure rate		
	5% higher	2.5% higher	Equal
Group-specific cure rates:			
Standard 12-week treatment groups	93.3%	95%	96.7%
Shortened treatment with ribavirin groups	98.3%	97.5%	96.7%
Shortened treatment without ribavirin groups	93.3%	92.5%	91.7%
Power for a:			
5% non-inferiority margin for regimen comparison	99%	98%	97%
10% non-inferiority margin for strategy comparison	100%	100%	96%
5% absolute difference for ribavirin comparison	98%	95%	91%

Group-specific cure rates are such that overall cure rate in 1092 patients is 95%, ribavirin effect between shortened treatment groups is 5% and there is no effect of regimen. The effect of ribavirin in shortened treatment compared with standard treatment is varied to reflect uncertainties in the cure rates and to vary the no effect of strategy between standard treatment and shortened treatment without ribavirin, shortened treatment with ribavirin and pooled shortened treatment groups. Power is calculated with one-sided alpha of 0.05 for non-inferiority margins and two-sided alpha of 0.05 for superiority comparisons reflecting the design. Power assumes that no groups have been stopped during recruitment.

Power to determine non-inferiority for the regimen comparison using a 5% margin is mostly unaffected by assumptions about different values for the standard 12-week cure rate and effect of ribavirin, and power remains close to 100%. Power to determine non-inferiority in the strategy comparison using a 10% margin is similarly unaffected, and power remains close to 100% when comparing the pooled shortening strategy groups against standard duration. For superiority comparisons, power to detect a 5% absolute difference in the ribavirin or regimen comparison remains high at more than 90% regardless of the standard duration cure rate and ribavirin effect.

### Limitations of the design

A potential weakness in the design is that the sample size was not originally calculated using Bayesian principles, but primary analyses will be conducted using Bayesian methods to allow for the calculation of posterior probabilities exploring the difference in cure rates between the interventions. However, for the non-inferiority comparisons, sample size estimates obtained using Bayesian methods are similar to or smaller than those obtained using frequentist methods [34], suggesting that our design is likely to be conservative. Additionally, secondary analyses will use frequentist methods for comparison. For interim analyses, the probability of correctly stopping a group, analogous to the frequentist concept of power, is determined by the true cure rate in the group and the number of analysed patients at each analysis and not by the overall group size.

The timing of and the number of patients at interim analyses are determined by at least one group, usually the 4-week treatment group with PEG-IFN since this has the shortest overall treatment duration, reaching a certain probability threshold of being stopped. This may mean delays in identifying unsuccessful groups receiving other strategies. This is unlikely if groups have cure rates lower than the average cure rate because, as discussed above, these will be detected faster than anticipated, but delays may occur if the cure rate is above the average but less than 90%. As the treatment length of patients in the RGT groups is unknown until after their day 7 visit, it is not possible to stagger treatment start dates so that the length between randomisation and EOT is the same for all strategies. Staggered treatment start might also lead to dropout after randomisation but before starting treatment, leading to inefficiency and potential bias. During the trial, cure rates will be monitored in all groups. If the cure rates are not as anticipated, either they are higher and lower than expected, and so our derived schedule is inappropriate based on these cure rates then the timing can be adjusted wih no penalty to the probability of incorrectly stopping recruitment into a randomised group, due to the use of Bayesian monitoring [22].

The power calculations for the final analysis assume that all groups will be included and that no groups have been stopped. It is possible that power will be lower if fewer groups are included, but for most comparisons with a full sample, power is very high and is likely to remain acceptable at the final analysis even with the exclusion of some patients. To help preserve power, if groups are stopped early subsequent patients will be randomly assigned to open groups. The power calculations were also estimated by using frequentist methods, although the primary analysis will use Bayesian methods. However, as power is extremely high, the analogous concept to power in Bayesian analysis, that for non-inferiority comparisons the lower credible interval bound is above the non-inferiority margin, is likely to be similarly high. Additionally, owing to the many possible combinations of strata and groups that could be stopped with different true and observed failure rates and at different times, examining the impact of stopping multiple combinations would require a large number of assumptions, probably also using a factorial simulation design, and hence would be a large piece of additional work in its own right. This is also the case for examining the impact of stopping multiple randomly assigned groups on other aspects of the trial, such as bias.

### Alternative designs

Alernative Bayesian designs, which have been used elsewhere [35], include basing the stopping guideline on a predictive probability, the probability of achieving a success at the end of the trial. In VIETNARMS, a success during the monitoring period is stopping a genuinely inferior group, which means that there is a more than 0.95 posterior probability of a less than 90% true cure rate in that group. A rule based on predictive probabilities would then state a group will be stopped at an interim analysis if there is a more than 0.95 chance of stopping a group at the end of the trial (Pr([Pr(true cure rate < 0.9|z) > 0.95]|x) > 0.95, where x is the data currently observed and z the complete data with all outcomes observed). For the monitoring beta (4.5, 0.5) prior and a fully recruited group, the stopping criteria are met with 13 failures, so equivalently the group is stopped if there is a more than 0.95 chance of observing at least 13 failures in the fully recruited group.

Predictive probabilities place a large emphasis on the arbitrary target cure rate of 90% and hence were not used for VIETNARMS. The final analysis will compare strategies against control and not test cure rates in individual groups. The aim of monitoring is to detect poorly performing groups and stop them early, rather than to ultimately meet a particular cure rate within a group at the end of the trial, as there may be other advantages to strategies that have a slightly lower cure rate than 90% in specific populations or circumstances. Compared with the posterior probability-based stopping guideline, using predictive probabilities requires a similar number of failures or more to stop a group (Supplementary Table 5, Additional file 1), so they do not offer any benefits in detecting poorly performing groups more quickly for our design, although they may for others [35]. Stopping rules and guidelines based on posterior probabilities can be converted to those based on predictive probabilities [36], so interim analyses can incorporate predictive probabilities to provide more information to the DMC.

Another approach would be to analyse the outcome data after every reported outcome rather than at scheduled interim analyses, which could reduce the time until a genuinely inferior group is stopped. Implementing this would be complex because of the many groups and varying treatment lengths. The small benefit in the reduction in time would not justify the additional work required to monitor outcomes intensely.

In VIETNARMS, if a group performs badly, then randomisation into that group will completely stop. Alternatively, the trial could have used response-adaptive randomisation where all groups would be retained but the allocation ratio would alter to favour randomisation into a group that is showing the most potential. For several reasons, this design was not considered suitable for VIETNARMS. First, each group will be tested separately and, as the randomisation is factorial, each group is the result of multiple randomisations and so it could be unclear how to adapt the randomisation allocations correctly. For example, the ribavirin groups may perform better with one shortening strategy but much worse with another strategy; in this case, it is unclear how the ribavirin randomisation allocation should be changed. Second, if a group is performing particularly badly and there is no prospect of that strategy being adopted, then continuing with that group would be a burden on resources, including on the number of patients enrolled. Third, in an open-label trial such as VIETNARMS, response-adaptive randomisation risks unblinding investigators to the relative performance of open groups, information that is usually privy only to a DMC.

## Conclusions

We have designed a trial allowing multiple approaches to drug choice and shortening strategy for HCV treatment to be simultaneously evaluated. We have closely examined the statistical aspects of the trial and focus particularly on the implications of the chosen rule for early stopping of unsuccessful individual groups. We have shown that the operating characteristics of the rule are appropriate and that interim analyses can be timed to detect individual groups that are highly likely to have suboptimal performance at various stages.

Given the pressures on funding and time, it is desirable to test many aspects of treatment at once and to allow for the swift removal of unsuccessful strategies: Bayesian monitoring methods allow for this. Despite the focus on HCV treatment, the statistical principles behind our novel design are not limited to this area and could be applied to other clinical trials in a wide variety of settings.

## Supplementary information

**Additional file 1: Supplementary Table 1.** Probability of stopping recruitment into a group. **Supplementary Table 2.** Predicted recruitment schedule. **Supplementary Table 3.** Sensitivity analysis of the timing of interim analyses comparing recruiting over 24 months to recruiting over 18 or 30 months. **Supplementary Table 4.** Sensitivity analysis of the timing of interim analyses altering the lower limit of the uniform distribution over which cure rates of genuinely inferior arms are assumed to be distributed. **Supplementary Table 5.** Sensitivity analysis comparing the use of a posterior probability-based rule to a predictive probability-based rule with a beta (4.5, 0.5) prior. **Supplementary Figure 1.** Cumulative probability of stopping interferon groups for different interim analysis schedules. **Supplementary methods.**

## Data Availability

Not applicable.

## Abbreviations

DAA: Direct-acting antiviral
DMC: Data monitoring committee
EOT + 12: 12 weeks after the end of treatment
HCV: Hepatitis C virus
LLOQ: Lower limit of quantification
MAMS: Multi-arm multi-stage
PEG-IFN: Pegylated interferon
RGT: Response-guided therapy
SVR12: Sustained virologic response 12 weeks after the end of treatment
VL: Viral load
WHO: World Health Organization
