# Supplementary material for: The design and statistical aspects of VIETNARMS: a strategic post-licensing trial of multiple oral direct-acting antiviral hepatitis C treatment strategies in Vietnam
Source: Trials. 2020 May 18;21:413. doi: 10.1186/s13063-020-04350-x (PMC7236096; doi:10.1186/s13063-020-04350-x)
Supplement: Supplementary file 1 — Additional file 1: Supplementary Table 1. Probability of stopping recruitment into a group. Supplementary Table 2. Predicted recruitment schedule. Supplementary Table 3. Sensitivity analysis of the timing of interim analyses comparing recruiting over 24 months to recruiting over 18 or 30 months. Supplementary Table 4. Sensitivity analysis of the timing of interim analyses altering the lower limit of the uniform distribution over which cure rates of genuinely inferior arms are assumed to be distributed. Supplementary Table 5. Sensitivity analysis comparing the use of a posterior probability-based rule to a predictive probability-based rule with a beta (4.5, 0.5) prior. Supplementary Figure 1. Cumulative probability of stopping interferon groups for different interim analysis schedules. Supplementary methods. [file 13063_2020_4350_MOESM1_ESM.docx]

**Additional file 1: Supplementary tables and figures**

Supplementary Table 1: Probability of stopping recruitment into a group

| True cure rate | Probability of stopping group | Probability of making correct decision | Probability of making incorrect decision |
| --- | --- | --- | --- |
| 60% | 1 | 1 | 0 |
| 70% | 0.999 | 0.999 | 0.001 |
| 80% | 0.877 | 0.877 | 0.123 |
| 82.5% | 0.740 | 0.740 | 0.260 |
| 85% | 0.530 | 0.530 | 0.470 |
| 87.5% | 0.312 | 0.312 | 0.688 |
| 90% | 0.138 | 0.138 | 0.862 |
| 92.5% | 0.040 | 0.960 | 0.040 |
| 95% | 0.008 | 0.992 | 0.008 |
| 97.5% | 0.001 | 0.999 | 0.001 |

Note: For true cure rates ≤90%, the correct action is to stop recruitment into a group; for cure rates >90% the correct action is to maintain recruitment into a group.

Supplementary Table 2: Predicted recruitment schedule

| Month since enrolment opened | Number recruited in the month | Total participants recruited | Total left to recruit |
| --- | --- | --- | --- |
| 1 | 8 | 8 | 1084 |
| 2 | 16 | 24 | 1068 |
| 3 | 24 | 48 | 1044 |
| 4 | 30 | 78 | 1014 |
| 5 | 35 | 113 | 979 |
| 6 | 40 | 153 | 939 |
| 7 | 52 | 205 | 887 |
| 8 | 52 | 257 | 835 |
| 9 | 52 | 309 | 783 |
| 10 | 52 | 361 | 731 |
| 11 | 52 | 413 | 679 |
| 12 | 52 | 465 | 627 |
| 13 | 52 | 517 | 575 |
| 14 | 52 | 569 | 523 |
| 15 | 52 | 621 | 471 |
| 16 | 52 | 673 | 419 |
| 17 | 52 | 725 | 367 |
| 18 | 52 | 777 | 315 |
| 19 | 52 | 829 | 263 |
| 20 | 52 | 881 | 211 |
| 21 | 52 | 933 | 159 |
| 22 | 52 | 985 | 107 |
| 23 | 52 | 1037 | 55 |
| 24 | 55 | 1092 | 0 |

Supplementary Table 3: Sensitivity analysis of the timing of interim analyses comparing recruiting over 24 months to recruiting over 18 months or 30 months

|  | First analysis | | | Second analysis | | | Third analysis | | | Fourth analysis | | |
| --- | --- | --- | --- | --- | --- | --- | --- | --- | --- | --- | --- | --- |
| Length of recruitment | 18 months | **24 months** | 30 months | 18 months | **24 months** | 30 months | 18 months | **24 months** | 30 months | 18 months | **24 months** | 30 months |
| Months since recruitment started | 6 | **7** | 8 | 8 | **10** | 12 | 11 | **13** | 17 | 14 | **18** | 25 |
| Total recruited | 255 | **205** | 136 | 375 | **361** | 266 | 555 | **517** | 471 | 780 | **777** | 841 |
| Total at EOT+12 weeks | 43 | **44** | 43 | 127 | **144** | 128 | 291 | **286** | 285 | 462 | **533** | 612 |
| At EOT+12 weeks in each: |  |  |  |  |  |  |  |  |  |  |  |  |
| PEG-IFN arm | 6 | **5** | 4 | 13 | **14** | 11 | 25 | **24** | 23 | 38 | **42** | 47 |
| RGT arm | 3 | **3** | 3 | 9 | **11** | 9 | 21 | **21** | 21 | 34 | **39** | 44 |
| I/M arm | 1 | **2** | 2 | 6 | **8** | 8 | 17 | **17** | 18 | 30 | **35** | 41 |

Note: the estimated time of recruitment (24 months) is in bold for clarity.

EOT+12: 12 weeks after the end of treatment; PEG-IFN: pegylated-interferon; RGT: response guided therapy; I/M: induction/maintenance
Note: assumes true cure rate in inferior arms is uniformly distributed over 60-90%.

Supplementary Table 4: Sensitivity analysis of the timing of interim analyses altering the lower limit of the uniform distribution over which cure rates of genuinely inferior arms are assumed to be distributed

|  | Lower bound of uniform distribution of cure rates of inferior arms | | | | | | | | |
| --- | --- | --- | --- | --- | --- | --- | --- | --- | --- |
| Probability of ≥1 arm being dropped | 0 | 0.1 | 0.2 | 0.3 | 0.4 | 0.5 | **0.6** | 0.7 | 0.8 |
| 30% | 5 | 5 | 7 | 7 | 8 | 9 | **10** | 13 | 18 |
| 40% | 7 | 7 | 7 | 8 | 9 | 10 | **11** | 15 | - |
| 50% | 7 | 7 | 8 | 9 | 10 | 11 | **13** | 17 | - |
| 60% | 8 | 9 | 9 | 10 | 11 | 13 | **15** | 24 | - |
| 70% | 9 | 10 | 10 | 11 | 13 | 15 | **18** | - | - |
| 80% | 11 | 13 | 15 | 16 | 17 | 23 | **-** | - | - |
| 90% | 18 | 23 | 22 | - | - | - | **-** | - | - |

Note: the lower bound used for the timing of analyses is in bold for clarity.

Supplementary Table 5: Sensitivity analysis comparing the use of a posterior probability based rule to a predictive probability based rule with a beta(4.5, 0.5) prior

|  | Stop if >95% posterior probability of true cure <90% | | | Stop if >95% predictive probability of >95% posterior probability of true cure rate <90% in fully recruited arm (i.e. 13 failures) | | |
| --- | --- | --- | --- | --- | --- | --- |
| Total at EOT+12 | Failures needed to stop (G) | P(observed failures ≥G \| true cure=89%) | P(observed failures ≥G \| true cure=90%) | Failures needed to stop (F) | P(observed failures ≥F \| 12 failures in fully recruited arm) | P(observed failures ≥F \| true cure=90%) |
| 3 | 3 | 0.001 | 0.00100 | 3 | 0.00339 | 0.00100 |
| 4 | 3 | 0.005 | 0.00370 | 3 | 0.01228 | 0.00370 |
| 5 | 3 | 0.011 | 0.00856 | 4 | 0.00195 | 0.00046 |
| 6 | 3 | 0.021 | 0.01585 | 4 | 0.00532 | 0.00127 |
| 7 | 3 | 0.033 | 0.02569 | 4 | 0.01127 | 0.00273 |
| 8 | 4 | 0.007 | 0.00502 | 4 | 0.02043 | 0.00502 |
| 9 | 4 | 0.012 | 0.00833 | 4 | 0.03331 | 0.00833 |
| 10 | 4 | 0.018 | 0.01280 | 5 | 0.00789 | 0.00163 |
| 11 | 4 | 0.026 | 0.01853 | 5 | 0.01319 | 0.00275 |
| 12 | 4 | 0.035 | 0.02564 | 5 | 0.02058 | 0.00433 |
| 13 | 4 | 0.046 | 0.03416 | 5 | 0.03043 | 0.00646 |
| 14 | 5 | 0.014 | 0.00923 | 5 | 0.04301 | 0.00923 |
| 15 | 5 | 0.019 | 0.01272 | 6 | 0.01189 | 0.00225 |
| 16 | 5 | 0.025 | 0.01700 | 6 | 0.01742 | 0.00330 |
| 17 | 5 | 0.032 | 0.02214 | 6 | 0.02465 | 0.00467 |
| 18 | 5 | 0.041 | 0.02819 | 6 | 0.03380 | 0.00642 |
| 19 | 5 | 0.050 | 0.03519 | 6 | 0.00928 | 0.00170 |
| 20 | 5 | 0.061 | 0.04317 | 6 | 0.01319 | 0.00239 |
| 21 | 6 | 0.022 | 0.01445 | 7 | 0.01826 | 0.00327 |
| 22 | 6 | 0.028 | 0.01822 | 7 | 0.02469 | 0.00439 |
| 23 | 6 | 0.034 | 0.02261 | 7 | 0.03267 | 0.00577 |
| 24 | 6 | 0.042 | 0.02766 | 7 | 0.04242 | 0.00746 |
| 25 | 6 | 0.050 | 0.03340 | 7 | 0.05412 | 0.00948 |
| 26 | 6 | 0.059 | 0.03986 | 7 | 0.06793 | 0.01187 |
| 27 | 7 | 0.024 | 0.01467 | 8 | 0.02195 | 0.00387 |
| 28 | 7 | 0.029 | 0.01791 | 8 | 0.02856 | 0.00495 |
| 29 | 7 | 0.034 | 0.02162 | 8 | 0.03660 | 0.00625 |
| 30 | 7 | 0.041 | 0.02583 | 8 | 0.04625 | 0.00778 |
| 31 | 7 | 0.048 | 0.03056 | 8 | 0.05770 | 0.00959 |
| 32 | 7 | 0.056 | 0.03585 | 8 | 0.07111 | 0.01169 |
| 33 | 7 | 0.064 | 0.04170 | 9 | 0.02257 | 0.00413 |
| 34 | 8 | 0.028 | 0.01686 | 9 | 0.02882 | 0.00513 |
| 35 | 8 | 0.033 | 0.01999 | 9 | 0.03638 | 0.00630 |
| 36 | 8 | 0.039 | 0.02351 | 9 | 0.04543 | 0.00767 |
| 37 | 8 | 0.045 | 0.027 | 9 | 0.056 | 0.009 |
| 38 | 8 | 0.052 | 0.032 | 9 | 0.069 | 0.011 |
| 39 | 8 | 0.059 | 0.037 | 9 | 0.083 | 0.013 |
| 40 | 8 | 0.067 | 0.042 | 9 | 0.100 | 0.016 |
| 41 | 8 | 0.075 | 0.048 | 10 | 0.032 | 0.006 |
| 42 | 9 | 0.036 | 0.021 | 10 | 0.040 | 0.007 |
| 43 | 9 | 0.042 | 0.024 | 10 | 0.049 | 0.009 |
| 44 | 9 | 0.047 | 0.028 | 10 | 0.061 | 0.010 |
| 45 | 9 | 0.054 | 0.032 | 10 | 0.074 | 0.012 |
| 46 | 9 | 0.061 | 0.036 | 10 | 0.089 | 0.014 |
| 47 | 9 | 0.068 | 0.041 | 10 | 0.107 | 0.016 |
| 48 | 9 | 0.076 | 0.046 | 10 | 0.127 | 0.019 |
| 49 | 10 | 0.038 | 0.022 | 11 | 0.037 | 0.008 |
| 50 | 10 | 0.043 | 0.025 | 11 | 0.046 | 0.009 |
| 51 | 10 | 0.049 | 0.028 | 11 | 0.057 | 0.011 |
| 52 | 10 | 0.055 | 0.032 | 11 | 0.070 | 0.013 |
| 53 | 10 | 0.061 | 0.035 | 11 | 0.085 | 0.014 |
| 54 | 10 | 0.068 | 0.040 | 11 | 0.103 | 0.017 |
| 55 | 10 | 0.075 | 0.044 | 11 | 0.124 | 0.019 |
| 56 | 11 | 0.040 | 0.021 | 11 | 0.148 | 0.021 |
| 57 | 11 | 0.044 | 0.024 | 11 | 0.175 | 0.024 |
| 58 | 11 | 0.050 | 0.027 | 12 | 0.041 | 0.011 |
| 59 | 11 | 0.055 | 0.031 | 12 | 0.051 | 0.013 |
| 60 | 11 | 0.061 | 0.034 | 12 | 0.064 | 0.015 |
| 61 | 11 | 0.068 | 0.038 | 12 | 0.080 | 0.017 |
| 62 | 11 | 0.074 | 0.042 | 12 | 0.098 | 0.019 |
| 63 | 11 | 0.081 | 0.047 | 12 | 0.122 | 0.021 |
| 64 | 12 | 0.045 | 0.024 | 12 | 0.150 | 0.024 |
| 65 | 12 | 0.050 | 0.026 | 12 | 0.184 | 0.026 |
| 66 | 12 | 0.055 | 0.029 | 12 | 0.224 | 0.029 |
| 67 | 12 | 0.060 | 0.033 | 12 | 0.273 | 0.033 |
| 68 | 12 | 0.066 | 0.036 | 12 | 0.332 | 0.036 |
| 69 | 12 | 0.073 | 0.040 | 12 | 0.402 | 0.040 |
| 70 | 12 | 0.079 | 0.044 | 12 | 0.485 | 0.044 |
| 71 | 12 | 0.086 | 0.048 | 12 | 0.583 | 0.048 |
| 72 | 13 | 0.049 | 0.025 | 12 | 0.700 | 0.053 |
| 73 | 13 | 0.054 | 0.028 | 12 | 0.838 | 0.058 |
| 74 | 13 | 0.059 | 0.031 | 12 | - | 0.063 |

Supplementary Figure 1: Cumulative probability of stopping interferon groups for different interim analysis schedules

Cumulative probabilities are assuming a a) true cure rate of 60% b) true cure rate of 70% c) true cure rate of 80%.

**Supplementary methods**

The model to be fitted in the logistic regression is:

$$\ln\left( \frac{p}{1-p} \right)= \beta_{0}+\beta_{1}\left( regimen \right)+\beta_{2}\left( strategy \right)+ \beta_{3}\left( ribavirin \right)+ \beta_{4}(genotype)$$

Where the options for regimen, strategy, ribavirin and genotype are listed in Figure 1. This model does not include the testing of interactions, which may not exist within the data.
